# Subjective and objective assessment of sedentary behavior among college employees

**DOI:** 10.1186/s12889-018-5630-3

**Published:** 2018-06-19

**Authors:** Samuel Headley, Jasmin Hutchinson, Sarah Wooley, Kristen Dempsey, Kelvin Phan, Gregory Spicer, Xanne Janssen, Jerold Laguilles, Tracey Matthews

**Affiliations:** 1 Department of Exercise Science and Sport Studies, Springfield College, Springfield Massachusetts, MA 01109 USA; 2 Cardiac Rehab/Non-invasive Cardiology, Newton-Wellesley Hospital, 2014 Washington St, Newton, MA 02462 USA; 3 University of Strathclyde, School of Psychological Science and Health, G1 1QE, Glasgow, Scotland UK; 4 Institutional Research, Springfield College, Massachusetts, Springfield, MA 01109 USA; 5 School of Health, Physical Education and Recreation Springfield College, Springfield Massachusetts, MA 01109 USA

**Keywords:** Occupational sitting, Transitions, Unbroken sitting, Academia

## Abstract

**Background:**

High levels of sedentary behavior are linked to increased mortality. In the United States, individuals spend 55–70% of their waking day being sedentary. Since most individuals spend large portions of their daily lives at work, quantifying the time engaged in sedentary behavior at work is emerging as an important health determinant. Studies profiling academic institutions, where a variety of personnel with diverse job descriptions are employed, are limited. Available studies focus mostly on subjective methods, with few using objective approaches. Therefore, the purpose of the current study was to assess sedentary behavior among all occupational groups of a college in the Northeastern United States utilizing both a subjective and an objective method.

**Methods:**

College employees (*n* = 367) completed the Occupational Sitting and Physical Activity Questionnaire (OSPAQ). A sub-sample of these employees (*n* = 127) subsequently wore an activPAL3 accelerometer 24 h per day for seven consecutive days. Outcome variables were time spent sitting, standing, stepping, and total number of steps. To assess fragmentation of sedentary behavior, the average duration of a sitting bout and sitting bouts/sitting hour were calculated. Differences between administrators, faculty, and staff, were analyzed using multivariate and univariate analyses of variance.

**Results:**

The OSPAQ results indicated that administrators spent more of their working day sedentary (73.2 ± 17.7%) than faculty members (58.5 ± 19.6%, *p* < 0.05). For the objective phase of the study, complete data were analyzed from 86 participants. During a waking day, administrators (64.0 ± 8.1%) were more sedentary than faculty (56.0 ± 7.9%, *p* < 0.05) and fragmented their sitting less than staff (3.7 ± 0.7 and 4.5 ± 7.9 bouts of sitting/sitting hour, respectively; *p* < 0.05). This pattern was also seen during working hours, with administrators (4.9 ± 2.1) taking fewer breaks per hour than staff (6.9 ± 3.0, *p* < 0.05).

**Conclusions:**

Administrators are the most sedentary members of the campus community. However, overall, the level of sedentary behavior among employees was high. This study highlights the need for sedentary behavior interventions in the college/university environment.

## Background

Sedentary behavior (SB) is defined as activities with which an individual is engaged while awake that result in an energy expenditure of < 1.5 metabolic equivalents (METS) while found in either a sitting, lying or reclining posture [1]. High levels of SB, independent of time spent in moderate-to-vigorous physical activity (MVPA), are linked to a host of adverse outcomes including increased rates of obesity, type 2 diabetes, colon cancer, increased levels of pro-inflammatory cytokines, decreased levels of anti-inflammatory markers and ultimately increased mortality [2–5]. Furthermore, extended time spent sitting without breaks for standing or walking, has been shown to be deleterious to health [6]. As little as 4 h (hrs) of cumulative sitting per day can have negative effects on a number of cardiometabolic risk factors [6]. Researchers have found evidence that in Western societies individuals spend 55–70% of their waking hours (9–11 h) being sedentary and for every additional hour spent sitting there is a 2% increase in the risk of mortality, even when MVPA is taken into account [5–8]. However, some evidence suggests that high levels of moderate intensity physical activity (60–75 min/day) can counteract the deleterious effects of prolonged periods of being sedentary [4, 5].

Due to the high prevalence of SB in modern society and the known adverse health effects associated with high levels of unbroken SB, the assessment of this component of our lives has become more important. Furthermore, since the largest percentage of the time spent sitting occurs at work, monitoring this behavior in the workplace has gained increased importance [5, 9]. Academic institutions seem suited for such studies since the nature of the work done in these institutions is mostly office based. In addition, academic institutions enable the examination of differences in SB between varied occupational roles (i.e. administrators, faculty and/or, staff).

Few studies have examined sedentary time, sedentary patterns (i.e. breaks in sedentary behavior) and/or differences in SB between employees at academic institutions. Using the Sedentary Behavior Questionnaire, Keenan & Greer [9] found that faculty members at a liberal arts university in the United States spent 7.9 ± 3.8 h per weekday being sedentary. In addition, Fountaine et al. [10] utilized the Occupational Sitting and Physical Activity Questionnaire (OSPAQ) to assess the level of SB at a regional university in Minnesota. The authors reported that members of the university community, including administrators, faculty, and staff members spent 5.8 ± 2.6 h (approximately 75%) of their working day being sedentary. A limitation of the aforementioned studies is the use of subjective assessments of SB. Subjective assessment provides information such as the type of behavior and perception of the behavior from an individual perceptive. Subjective assessments include paper and pencil instruments, interviews, and online tools. Kang and Rowe [7] reported that subjective measures of sedentary behavior are helpful to determine the type of behavior that occurs in larger observation studies and these measures can be more practical and efficient; however, there are limitations to subjective assessment of sedentary behavior. Since sedentary behavior occurs throughout the entire day, it may be more difficult for individuals to accurately recall the amount of time spent in sedentary behavior [7]. An objective assessment is observable and directly measurable, for example an accelerometer or pedometer. Two studies using objective measures of SB reported that university employees spent 68% [8] and approximately 72% [6] of their working day being sedentary. However, these studies did not report on specific patterns of SB and only one of the two studies examined differences in SB according to occupational role [6, 8].

To inform future interventions focusing on SB in the office environment and policy development, understanding both the levels of SB and patterns of SB are important. Since there is a paucity of objective information regarding the amount of SB and no evidence on the patterns of SB among employees at academic institutions in the United States, the purpose of the current study was to accurately document the amount and patterns of SB at work among all employee groups at a private 4-year college in the Northeastern United States. In addition, differences in SB between occupational roles were examined. Occupational roles at this institution are delineated into three categories by the Human Resource Office: administrator (any person in a management position but who does not teach students), faculty (those involved with teaching students), and staff (employees who support the work of the administrators but who do not teach). The level of SB and pattern of SB within these specific job roles was examined to determine if there were differences in SB between job roles. If so, this examination might provide objective data that could later be used to target certain occupational categories and customize more effective interventions for these job roles. It was hypothesized that employees classified as staff and administrators would spend more time engaged in SB than faculty due to the nature of the jobs performed by each of these groups.

## Methods

### Participants

All employees, at a college in the Northeast United States, noted for its rich history in physical education and exercise science, received emails and flyers about the study during the beginning of the fall semester (i.e., September/October 2016). Individuals were eligible to participate in the study if they were at least 18 years old and were classified as full-time employees at the institution. Participants self-reported their occupational classification (administrator, faculty, or staff) when providing consent and this was verified by consultation with Human Resources if there was any ambiguity. The study was reviewed and approved by the Institutional Review Board of the College and data were only collected on individuals who gave their informed consent to participate.

### Subjective SB measurement

Eligible employees were asked to complete an online version of the OSPAQ [11] a self-report instrument to measure perceptions of behavior. The OSPAQ asked participants to recall how many hours they spent at work and what percentage of time they spent sitting, standing, walking and doing physically demanding tasks at work in the previous 7 days. These data were then transformed into minutes (mins) spent in each category per working day. The OSPAQ has been found to be a moderately reliable tool to assess SB [12]. The survey was distributed to all employees via email in September 2016 and remained open for 4 weeks with weekly prompts being sent out to remind the different constituents of the College to complete the survey.

### Objective SB measurement

The College in this study has a number of regional campuses across the country. All College employees at all campuses received the electronic OSPAQ survey, but due to logistical reasons (satellite campuses are located in seven different states across the country) only employees at the main campus were invited to participate in the second (objective) phase of the study. A sub-sample of members of the main campus community (*n* = 127) expressed interest in participating in the second phase of the study, which involved the measurement of SB using an activPAL3 (PAL Technologies, Glasgow, UK) accelerometer. Data collection followed good practice guidelines as recently published by Edwardson et al. [11]. Participants were asked to wear the activPAL3 for 7 days. The activPAL3 was placed in a nitrile fingercot (a small water impermeable sheath) and attached to the midline anterior aspect of the upper thigh using Tegaderm dressing (3 M, Minnesota, USA) as recommended by the manufacturer. Participants were told that they could shower with the device in place, but they should not be immersed in water or participate in any contact sports. In addition, participants were asked to complete a diary recording their bed (i.e. “lights out”) and wake times as well as the times they started and finished work each day. For this objective measurement part of the study, participants were included in the waking day analysis if they provided a completed wear time diary and at least 3 days of valid wear (i.e. 3 days with more than 600 mins of wear per day) and in the work day analysis if they provided at least 3 complete working days of valid wear (i.e. 3 days with > 75% valid wear based on self-reported working hrs).

ActivPAL3 event files were created using the activPAL software provided by the manufacturer and using a minimum of 10 s for the sitting/upright period as recommended by Edwardson et al. [11]. A specialized macro (available from XJ upon request) was then used to identify waking day hours and working day hours based on the participants’ diaries. This information was then used to compute outcome variables, which were minutes per day spent sitting, standing, or stepping as well as the average % of time spent in these behaviors during the whole day and during a working day (to account for differences in wake and work times). In addition, to measure the fragmentation of SB (i.e. the extent to which sedentary behavior is prolonged or interrupted) the number of bouts in SB per sedentary hour and the average length of time individuals spent in bouts of SB were calculated.

### Statistical analysis

Descriptive analyses were conducted on the OSPAQ and activPAL data. Differences between participant characteristics and differences in time spent being sedentary between different occupational groups measured using the OSPAQ were analyzed using one-way ANOVA analyses. MANOVA analyses were used to analyze differences in time spent being sedentary between different occupational groups for the activPAL data using SPSS (version 24 IBM Corp, Armonk, NY). The level of statistical significance was set at *p* < 0.05.

## Results

All data are presented as means ± SD. The OSPAQ was sent to 697 college employees of whom 367 (52.7%) provided complete data that was used in our analyses. From the total campus community at the main campus (*n* = 580), 127 (21.9%) volunteered to have an objective assessment made of their activity for 7 consecutive days using an activPAL3 accelerometer. Three of these individuals reported having a rash at the site of attachment of the accelerometer that was therefore removed. Another 38 individuals did not provide complete diaries to note their working day or sleep time and were excluded from the analysis. Therefore, analyses were performed on data collected from 86 individuals. The physical characteristics of these 86 individuals can be seen in Table 1. Participants in all three groups did not differ in age, height or BMI. However, participants in the administrator group had significantly higher weight compared to those in the staff group (*p* < 0.05), and faculty had significantly lower waist circumference than both administrators and staff (*p* < 0.05).

**Table 1:** Physical characteristics of employees who completed valid assessments (mean, SD)

Variable	All	Administrators	Faculty	Staff
N (% male)	86 (72.9)	22 (59.1)	25 (76.0)	39 (76.9)
Age (years)	48.48 (11.39)	51.95 (9.08)	47.84 (11.63)	46.92 (11.39)
Height (cm)	165.7 (8.7)	165.4 (8.0)	164.3 (9.7)	166.7 (8.5)
Weight (kg)	80.2 (18.8)	86.7 (17.9)^a^	72.6 (16.6)^a^	81.3 (19.4)
Waist (cm)	91.1 (15.0)	96.7 (12.1)^a^	83.0 (12.5)^a,b^	93.2 (16.1)^b^
BMI (kg/m^2^)	29.2 (0.4)	31.7 (5.9)	27.1 (7.6)	29.2 (6.2)

^a^Administrators <> Faculty, *p* < 0.05

^b^Faculty <> Staff, *p* < 0.05

The objective data that are presented in Tables 2 and 3 represent information that was collected from three occupational groups; administrators (*n* = 22), faculty (*n* = 25) and staff (*n* = 39). Detailed results for the whole day are described in Table 2, There was a statistically significant difference in time spent sedentary (%) based on the job role, F(4, 164) = 3.255, *p* < 0.05; Wilk’s Λ = 0.858, partial η^2^ = 0.074. Post-hoc analyses revealed administrators spent significantly more time sitting compared to faculty (mean difference 8.1%, *p* < 0.05) and significantly less time stepping per day (mean difference 2.5%, *p* < 0.05). For the fragmentation of sedentary time (i.e. the extent to which sedentary behavior is prolonged or interrupted) there was a significant difference based on the job role, F(4, 164) = 3.284; Wilk’s Λ = 0.857, partial η^2^ = 0.074. Post-hoc analyses revealed no differences in the average duration of sitting bouts, however, administrators recorded fewer sedentary bouts per sedentary hour compared to staff (mean difference 0.83 bouts/sedentary hour, *p* < 0.05), meaning administrators broke up their sitting less. A statistically significant difference in time spent stepping (% and mins) based on the job role was observed, however the follow-up post-hoc tests were non-significant. The number of steps taken per day were not significantly different between groups (*p* > 0.05).

**Table 2:** Total day sedentary behavior data collected using the ActivPAL3 (mean, SD)

	Whole day				
	*All*	*Admin*	*Faculty*	*Staff*	p-value*
Valid days	5.9 (0.4)	5.8 (0.4)	5.9 (1.9)	6.0 (1.9)	0.112
Wear time (hrs/day)	15.8 (1.0)	16.1 (0.8)	15.7 (1.1)	15.7 (1.0)	0.245
Sedentary time (%)	59.4 (9.3)	64.0^a^ (8.1)	55.9^a^ (7.7)	59.0 (10.2)	0.010
Sedentary time (mins)	563.1 (96.4)	618.0^a,b^ (78.4)	526.4^a^ (75.7)	555.8^b^ (108)	0.003
Standing (%)	29.0 (8.3)	25.4 (6.1)	31.0 (7.6)	29.8 (9.3)	0.047
Standing (mins)	275.0 (79.1)	246.0 (62.6)	292.0 (80)	281.2 (86.6)	0.112
Stepping (%)	11.6 (3.3)	10.6^a^ (3.8)	13.1^a^ (3.1)	11.2 (2.9)	0.021
Stepping (mins)	110.3 (31.6)	102.5 (35.5)	124.3 (31.3)	105.4 (28.2)	0.038
Total steps	8810.7 (2833.6)	8429.9 (3346.5)	9817.2 (2723.9)	8380.4 (2478.9)	0.107
Number of SB bouts/SB hour	4.2 (1.0)	3.7^b^ (0.7)	4.3 (1.1)	4.5^b^ (0.9)	0.003
Average length of sedentary bout (mins)	16 (4.2)	17.7 (3.7)	15.5 (5.1)	15.3 (3.7)	0.085

*p-values for MANOVA between subjects tests or ANOVA test (total steps, wear time and valid days)

^a^Administrators <> Faculty, *p* < 0.05

^b^Admin <> Staff, *p* < 0.05

**Table 3:** Work day sedentary behavior data collected using the ActivPAL3 (mean, SD)

	Work day				
	*All*	*Admin*	*Faculty*	*Staff*	p-value*
Valid days	4.1 (1.0)	4.3 (0.9)	4.4 (0.9)	3.9 (1.0)	0.206
Wear time (hrs/day)	8.0 (8.0)	8.2 (1.2)	7.6 (1.6)	8.2 (0.8)	0.079
Sedentary time (%)	57.0 (15.7)	60.8 (15.3)	53.2 (11.5)	57.3 (17.9)	0.251
Sedentary time (mins)	275.6 (90.0)	299.3 (85.9)	240.4 (72.4)	284.9 (97.4)	0.054
Standing (%)	31.7 (14.7)	29.2 (15.0)	33.2 (10.1)	32.2 (17.1)	0.625
Standing (mins)	151.7 (70.8)	143.0 (72.8)	151.0 (55.5)	157.1 (79.1)	0.761
Stepping (%)	11.3 (5.6)	10.0 (5.4)	13.6 (6.9)	10.5 (4.5)	0.046
Stepping (mins)	54.0 (26.6)	48.8 (24.1)	62.6 (34.0)	51.5 (21.5)	0.148
Total steps	4684.8 (2361.0)	4443.3 (2369.4)	5162.6 (2769.1)	4514.8 (2076.1)	0.488
Number of SB bouts/SB hour	5.8 (2.6)	4.9^a^ (2.1)	5.2^b^ (1.9)	6.7^a,b^ (3.0)	0.011
Average length of sedentary bout (mins)	13.9 (8.2)	14.8 (6.0)	15.3 (9.6)	12.6 (8.3)	0.387

*p-values for MANOVA between subjects tests or ANOVA test for total steps, wear time and valid days;

^a^Faculty <> Staff, *p* < 0.05

^b^Admin <> Staff, *p* < 0.05

Detailed results for the workday are described in Table 3. There was no statistically significant difference in time spent sedentary (%) based on the job role, F(4, 162) = 1.816, *p* = 0.128; Wilk’s Λ = 0.917, partial η^2^ = 0.042.

In addition, for the fragmentation of sedentary time, there was a significant difference based on the job role, F(4, 162) = 2.535, *p* < 0.05; Wilk’s Λ = 0.887, partial η^2^ = 0.058. Post-hoc analyses revealed no differences in the average duration of sitting bouts, however, staff recorded more sedentary bouts per sedentary hour compared to administrators and faculty (mean difference 1.81 bouts/sedentary hour and 1.51 bouts/sedentary hour, respectively, *p* < 0.05), meaning staff broke up their sedentary time more during the work day compared to administrators and faculty. The steps taken at work were not different between the groups with all groups recording values in the 4000–5000 steps per day range.

Based upon the subjective data collected using the OSPAQ, the administrators (73.2 ± 17.3%) spent more of their workday sitting compared to the faculty (58.6 ± 19.9%, *p* < 0.05), however, administrators did not differ from staff (68.2 ± 24.1%, *p* = 0.237) (Table 4).

**Table 4:** Subjective data collected using the OSPAQ (mean, SD)

Variable	All	Administrators	Faculty	Staff
Workday sitting (mins)	327.7 (168.3)	357.9 (137.5)	323.8 (203.4)	314.3(151.2)
Workday sitting (%)	66.2 (21.9)	73.2 (17.3)^a^	58.9 (19.9)	68.2 (24.1)
Workday standing (mins)	89.4 (89.2)	61.8 (53.5)	137.1 (99.9)^b^	66.9 (80.6)
Workday standing (%)	17.6 (15.5)	12.5 (9.5)	25.6 (14.8)	14.1 (16.3)

^a^Administrators > Faculty, *p* < 0.05

^b^Faculty > Administrators and Staff, *p* < 0.05

Staff spent more time of their workday sitting compared to faculty (*p* < 0.05). The OSPAQ data also showed that the faculty spent more of their workday standing (137.1 ± 99.9 mins) than either the administrators (61.8 ± 53.5 mins) or staff (66.9 ± 80.6 mins, *p* < 0.05) (Table 2).

## Discussion

The primary purpose of the current study was to assess SB across all constituents of the professional full-time staff at a 4-year college in the Northeast United States. Our major findings were that the college employees spent a large amount (between 8.5 to 10.5 h per day or 55–65%) of their day being sedentary. Both during the whole day (i.e., work and non-working hours) and when at work, the administrators took fewer breaks from sitting than the staff. Furthermore, during working hours, staff took more breaks from sitting than the faculty. Our findings provide objective data to indicate a need to develop targeted interventions aimed at encouraging all college employees, particularly administrators to reduce their sedentary time and to alter their pattern of sedentary behavior. This is important given the fact that engaging in prolonged periods of unbroken sedentary behavior is associated with poor health outcomes.

A major strength of the current study is the use of the activPAL3 device to objectively measure SB. Previous researchers have found the activPAL to be accurate in measuring SB due to its capability as a posture-based monitor [12–15]. Additionally, the activPAL has been shown to be a sensitive tool in detecting changes in SB [14, 16, 17]. There have only been a few reports in the literature regarding the assessment of SB among individuals working at academic institutions [6, 8–10]. Two of these studies used self-reported online surveys like the OSPAQ to assess SB [9, 10]. The current study is the first to objectively assess SB across all employee groups within an academic institution in the United States. Urda et al. [8] assessed SB among 44 female office workers at Slippery Rock University and reported that these female workers spent 68% of their workday being sedentary. The higher percentage of reported time spent sedentary time by Urda et al. may be due to them including only participants whose job profiles were very sedentary. In contrast, the current study included all employee groups including those whose job profile would be slightly less sedentary. Similar to what was done in the current study, Bird et al. [18] assessed SB and physical activity levels across all segments of the university community at the University of Tasmania and reported that their participants (*n* = 15) spent 71.5 ± 13.1% of their workday being sedentary. In the current study, across all employee groups during the working hours, the average proportion of the day spent being sedentary was 56.9 ± 15.8% which is lower than that reported by Bird et al. [6] reported. Differences may be due to the measurement tool used. Bird et al. used a SenseWear monitor to assess SB and physical activity. The SenseWear monitor is not a posture-based monitor and may include upright activities such as standing still or a very slow walk as SB [19].

It is also important to highlight the fact that the sample sizes in both of the objective studies cited were less than what we report here, and the current study is more inclusive of members from the campus community [6, 8]. Since our sample includes data from all segments of the campus community we have been able to compare SB among these various groups. Consequently, we have noted that the administrators are the most sedentary employee group, something that has not been previously reported. We have also noted that the staff members broke up their sedentary time more than other groups and that the administrators were less likely to break up their sedentary time. Researchers have found that breaking up prolonged sitting has several beneficial effects while unbroken sitting has detrimental physiological effects [14, 15]. Unbroken sitting leads to endothelial dysfunction which is thought to be mediated by reduced sheer stress [16]. This endothelial dysfunction is a precursor to the development of atherosclerosis which is known to be the underlying mechanism for the development of cardiovascular disease; the major cause of death in developed countries [16]. Even small amounts of leg movement, (e.g. fidgeting) has been shown to prevent the endothelial dysfunction induced by prolonged unbroken sitting [20]. There is therefore a need for the administrators at this institution, to reduce the overall time that they spend being sedentary and to include more frequent breaks from sitting in their day both at work, and when they are away from work. By doing so they are likely to reduce the cumulative deleterious physiological effects of prolonged unbroken sitting.

Though the focus of the current study was to assess the level of SB objectively, it was interesting to note that the subjective assessment of SB painted a similar picture to the one that was captured using the objective measures. Furthermore, the data from the OSPAQ instrument supported the findings from the use of the activPAL devices that the employees at this institution were less sedentary than what has previously been reported by Fountaine et al. [10]. A possible explanation for this could be the fact that this particular institution has a rich history in physical education and exercise science and a culture of high levels of physical activity is nurtured at this institution [21]. The latter assertion is supported by the average number of steps registered during the course of the whole day across all employee groups which was well above the 7000 steps per day as recommended by the American College of Sports Medicine [22, 23].

This study had some limitations. The sample was size relatively low for an observational sample. In addition, the use of the activPAL3 device may have resulted in some reactivity of participants due to awareness of being monitored. However, several studies have shown reactivity to wearable technology is relatively low if not non-existent [24, 25]. Due to logistical reasons, we could not collect demographic data on employees who participated in the subjective assessment phase of the study. Last, there was a low participation rate among members from the facilities management department who, by nature of their jobs, spend most of their working hours performing physical tasks. It is therefore likely that the rates of SB that we report among staff members would have been lower if more members from the facilities management department had participated.

Due to the high prevalence of “desk-jobs” within academia, the risk of engaging in SB seems high in this workforce and could be regarded as an occupational hazard that needs to be addressed [6]. However, without a clear assessment of the current levels of SB there would be no feasible way of gauging the effectiveness of interventions that are designed to address this emerging problem. The current study was specifically designed to provide that objective information that will be used to design future interventions to tackle this issue. Based upon our findings, interventions are needed to specifically target administrators, to encourage them to reduce their SB both at work and away from work and to intentionally include more breaks from sitting in their day. Since administrators are the decision makers at the institution, interventions that successfully impact upon this group could have a “trickle down” effect that could positively impact other constituents of the institution since they could implement policies and allocate resources to reduce campus wide SB. An intervention could take the form of targeted messaging (i.e. prompts) to these employees to encourage them to take regular breaks from sitting and suggest some simple strategies such as standing while taking calls, using a bathroom on a different floor, and placing printers and photocopiers in common areas where an individual has to make a trip away from their work area in order to use them. The fact that the administrators tended to weigh more and to have higher waist circumferences than other employee groups highlight the urgent need for such interventions.

## Conclusions

Administrators were the most sedentary group both at work and away from work.

The staff tended to break up their sedentary time more than other employee groups at work.

## Abbreviations

METS: Metabolic equivalent
MVPA: Moderate to vigorous physical activity
OSPAQ: Occupational Sitting and Physical Activity Questionnaire
SB: Sedentary behavior
